# Joint effect of elevated-c-reactive protein level and hypertension on new-onset stroke: A nationwide prospective cohort study of CHARLS

**DOI:** 10.3389/fpubh.2022.919506

**Published:** 2022-10-03

**Authors:** Xuanli Chen, Siyuan Liu, Jiadong Chu, Wei Hu, Na Sun, Yueping Shen

**Affiliations:** Department of Epidemiology and Biostatistics, School of Public Health, Medical College of Soochow University, Suzhou, China

**Keywords:** joint effect, C-reactive protein, hypertension, stroke, China Health and Retirement Longitudinal Study

## Abstract

**Background and aims:**

This study aimed to examine whether the combination of elevated-C-reactive protein (CRP) levels and hypertension increased the risk of stroke among middle-aged and elderly Chinese.

**Methods:**

This analysis included 9,821 Chinese participants aged ≥45 years in the China Health and Retirement Longitudinal Study (CHARLS). Data based on three waves of CHARLS were used (2011, 2013, and 2015). Multivariable Cox proportional hazards regression models were used to estimate hazard ratios (HRs) with a 95% confidence interval (95%CI) of new-onset stroke risk according to elevated-CRP level and hypertension. Moreover, the area under the curve (AUC), net reclassification index (NRI), and integrated discrimination improvement (IDI) were used to evaluate the incremental predictive value.

**Results:**

A total of 184 stroke events occurred during follow-up. The median follow-up time was 4 years. Compared with those with normal CRP levels (CRP ≤ 3 mg /L) and blood pressure, the adjusted HRs and 95%CI were 1.86 (0.90–3.85) for individuals with elevated-CRP levels alone, 2.70 (1.71–4.28) for those with hypertension alone, and 4.80 (2.83–8.12) for those with comorbid elevated-CRP levels and hypertension. People with the coexistence of elevated-CRP levels and hypertension had the highest risk of new-onset stroke among all subgroup analyses. Finally, adding the combination of elevated-CRP levels and hypertension to conventional factors significantly improved the risk prediction for new-onset stroke.

**Conclusion:**

Our findings indicate that the combined effect of elevated-CRP levels and hypertension increase the risk of new-onset stroke among the middle-aged and geriatric Chinese population.

## Introduction

As per recent statistics, stroke was the second largest cause of death worldwide and the second most common cause of global disability-adjusted life years (DALYs) (1, 2). According to the Heart Disease and Stroke Statistics-−2020, there has been a decline in stroke age-standardized mortality and DALYs over the past several decades; however, the decrease in age-standardized incidence has been less steep (2), indicating that the burden of stroke is likely to remain high. The same report showed that in China (2), the stroke age-standardized incidence has increased in China, contrary to its global decline (3). Thus, these statistics indicate the immediate need for more effective preventive and control measures.

Previous studies indicate that C-reactive protein (CRP) is elevated in individuals who are at risk of developing coronary artery disease or undergoing adverse cerebrovascular events. This elevation can be noted many years prior to the detection of vascular disease (4). CRP is an acute-phase reactant and prototypic downstream marker of inflammation and is a part of the innate immune response. It is mainly produced in the liver under the stimulation of IL-6 (5). Since it is an indicator of systemic inflammation, CRP may predict the burden of atherosclerosis and has a predictive and diagnostic role in different types of stroke, such as ischemic stroke and fatal stroke (4, 6), but the role of CRP in predicting hemorrhage stroke outcome was even less clear (7). Hypertension is a well-recognized modifiable risk factor for stroke (8–10). It affects approximately half the Chinese population in the 35–75 years age group, as per the China Patient-Centered Evaluative Assessment of Cardiac Events (PEACE), and 84.2% of stroke survivors suffer from hypertension (11, 12). Hypertension accentuates the progression of atherosclerosis, which is the most common cause of stroke (13). Elevated-CRP levels and hypertension may be positive factors in the progression of atherosclerosis. Therefore, we believe that the co-occurrence of both conditions can increase the risk of new-onset stroke by accelerating the progression of atherosclerosis.

Few studies have evaluated the combined effect of elevated-CRP levels and hypertension on the risk of new-onset stroke in the general population. Therefore, we conducted a prospective study in 450 Chinese communities based on the China Health and Retirement Longitudinal Study (CHARLS) to examine the combined effects of elevated-CRP levels and hypertension on stroke risk.

## Patients and methods

### Study design and population

The present study was ancillary to the China Health and Retirement Longitudinal Study (CHARLS), an ongoing, nationwide cohort study of the Chinese population aged ≥45 years, to assess the social, economic, and health status (14). The sample for CHARLS was obtained from 450 communities within 150 districts and 28 provinces through multistage probability sampling (14), and 10,257 households participated with 17,708 individuals in the baseline survey (15), respectively. The CHARLS study protocol was approved by the ethics review committee at Peking University, Beijing, China, and written informed consent was obtained from all participants.

**Figure 1:**
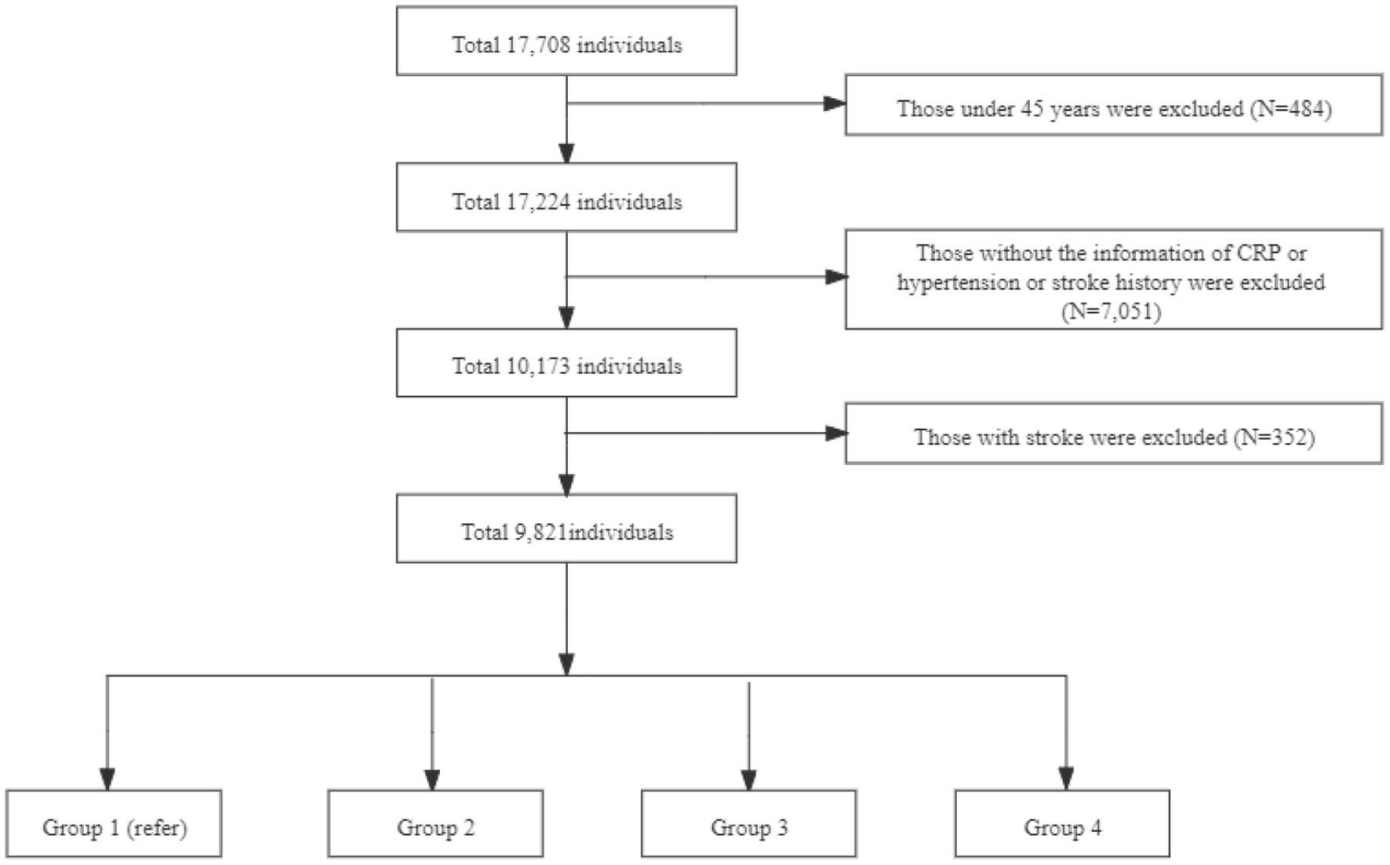
Flowchart of participant selection in CHARLS.

Note: Group 1 is non-high c-reactive protein (HCRP) and non-hypertension with 4,462 individuals; Group 2 is HCRP and non-hypertension with 785 individuals; Group 3 is non-HCRP and hypertension with 3,592 individuals; Group 4 is HCRP and hypertension with 982 individuals.

This study used data from 2011 to 2015, including one baseline survey (2011–2012) and two follow-up waves (2013, 2015). We excluded individuals aged <45 years, had incomplete information of CRP concentration and hypertension, a history of stroke, without a history of stroke information at baseline, and did not follow up. The final sample consisted of 9,821 individuals (Figure 1). Participants were categorized into four groups according to CRP levels and hypertension: (a) Group 1: Normotensive individuals with normal CRP levels (CRP ≤ 3 mg/L); (b) Group 2: Normotensive individuals with elevated-CRP levels (CRP >3 mg/L); (c) Group 3: Hypertensive individuals with normal CRP levels; (d) Group 4: Hypertensive individuals with elevated-CRP levels.

### Serum elevated-CRP level assessment

The 2011–2012 national baseline blood data users' guide shows that CHARLS has successfully collected and analyzed venous blood samples from a large population. The elevated-CRP level was defined as participants as those with a high sensitivity CRP of >3 mg/L (16). Normal CRP levels were defined as high sensitivity CRP of ≤ 3 mg/L. The high sensitivity CRP was measured with venous blood samples by immunoturbidimetric assay in the Clinical Laboratory of Capital Medical University from frozen plasma or whole blood samples. The detection limit of high sensitivity CRP was 0.1–20 mg/L, the within assay of high sensitivity CRP coefficient of variation was <1.3%, and between assay was <5.7%.

### Hypertension assessment

The participants were defined as having hypertension when their SBP (systolic blood pressure) was ≥140 mmHg and DBP (diastolic blood pressure) was ≥90 mmHg. Alternatively, participants with a self-reported history of hypertension or participants who have used antihypertensive drugs were also considered hypertensive (17). Normotensive participants were defined as individuals whose SBP was <140 mmHg, DBP was <90 mmHg, without a self-reported history of hypertension, and without prior use of any antihypertensive drugs, all the above standards were satisfied. Trained staff measured the participants' blood pressure on the left arm in a sitting position after resting for at least 10 min. The mean value of the two blood pressure measurements was used for data analysis.

### New-onset stroke and onset time of stroke assessment

A stroke event was defined as a new-onset stroke that occurred during the follow-up by a self-reported model (18). The trained staff asked the participant the following survey questions: (i) Have you been diagnosed with stroke by a doctor? (ii) When was the condition first diagnosed/known by yourself? If an affirmative answer is determined by the individual at follow-up, then the participant would be classified as having their first stroke and the self-reported time was noted as the onset time of stroke. Special conditions were employed to improve the accuracy of estimation of the onset time of stroke, as the exact time of stroke development was not available for all participants. They were considered as follows: First, if the participants did not develop stroke in any of the follow-up wave surveys (the time to event was calculated as follows: the time of the last survey—the time of baseline investigation); the follow-up time was not available (the approximate estimated time to event is defined as follows: the integer number years of the time of the last survey—the time of baseline investigation). Second, if they developed stroke (the time to event was defined as: the time of specific wave with stroke information/2—the time of interval wave/2 + the time of interval wave—the time of baseline investigation); the follow-up time was not available (the approximate time to event is defined as the integer number years of the time of specific wave with stroke information/2—the time of interval wave/2 + the time of interval wave—the time of baseline investigation).

### Other covariates assessments

Covariates, including age, sex, self-reported education level, living place, annual per-capita income, self-reported smoking, and drinking status, BMI (body mass index), basic ADL (Activities of Daily Living Scale), instrumental ADL, and self-reported history of dyslipidemia, diabetes, high blood sugar, heart diseases, cancer, chronic lung disease, memory-related disease, kidney disease, liver disease, arthritis, digestive disease, asthma, and psychiatric problems were also measured in the baseline survey. Education level was categorized as: less than lower secondary, upper secondary and vocational training, and tertiary level (19). The type of living place was classified as an urban community and a rural village. Annual per-capita income (API) was categorized into three: poverty (<2,800¥), low-income (2,800 ≤ API ≤ 10,000¥), and high-income (API ≥ 10,000¥) (19). Smoking and drinking status were classified into ever or never (14). BMI was calculated by dividing the individual's weight by their height squared (kg/m^2^) (20). The basic ADL included six items: dressing, bathing, eating, getting out of bed, using the toilet, controlling urination, and defecation, with a score of 0–6, wherein 0 indicated not having any difficulty in any of the six items. The basic ADL score was classified into good (0) or bad (>0) (21). Instrumental ADL includes five items: performing household chores, preparing hot meals, shopping, managing assets, and taking medications (21). The value range of basic ADL is 0–6 [good (0) or bad (>0)], wherein 0 indicated not having any difficulty in any of the five items. The self-reported history of specific diseases included general diseases, except for tumors or cancer. The term self-reported history of cancer recorded detailed information about cancer or tumor (18).

### Statistical methods

Median (lower quartile-high quartile), mean and standard deviation (mean ± SD), and frequency (percentage) were used for the description of continuous and categorical variables, respectively. The difference between categorical variables was evaluated by the Pearson Chi-squared test, and differences between continuous variables by the Kruskal–Wallis test or analysis of variance were used for the four groups. Kaplan–Meier curves and the log-rank test were used to compare the cumulative risk of events according to elevated-CRP levels and hypertension. Multivariable Cox proportional hazards regression was used to estimating hazard ratios (HRs) and 95% confidence intervals (95%CI) between the exposure of elevated-CRP levels, hypertension, and new-onset of stroke. The Cox model met the proportional assumption (*P* = 0.9218). The area under the curve (AUC), net reclassification index (NRI), and integrated discrimination improvement (IDI) were used to evaluate the incremental predictive value. The adjustment for confounding factors included demographic characteristics, lifestyle factors, and chronic disease variables. We also performed a series of sensitivity analyses to check the robustness of findings, including the other definition of hypertension; individuals develop hypertension during follow-up; different stage hypertension (22). The subgroup analyses were performed to evaluate the combined effect of elevated-CRP levels and hypertension on new-onset stroke, considering age, sex, living place, and BMI. The statistical data analysis software package SAS 9.4 (SAS Institute Inc., Cary, North Carolina, USA) was used for all the data analyses. The statistical significance level was set as 0.05 (two-tailed).

## Results

### Characteristics of the participants

Baseline characteristics of the study population of different groups are depicted in Table 1. The final sample consisted of 9,821 participants during the 4-year follow-up and the prevalence of new-onset stroke was 1.87% (*N* = 184). Compared with the coexistence of low CRP and non-hypertension, the prevalence of high CRP levels along with simultaneous hypertension was noted to be more common among females at baseline. Furthermore, individuals with high CRP and simultaneous hypertension were older, smokers, living in urban localities, had lower income, poorer basic ADL scores and instrumental ADL scores, poor marital status, and a higher BMI. The prevalence of nearly all chronic diseases (except liver, kidney, and psychosis-related diseases) showed significant differences among the four groups (all *P* < 0.05).

**Table 1:** Characteristics of the study population in four groups in CHARLS.

**Variables (%)**	**All participants**	**Group 1^a^**	**Group 2^b^**	**Group 3^c^**	**Group 4^d^**	***P-*value^e^**
*N*	9,821	4,462 (45.43)	785 (7.99)	3,592 (36.57)	982 (10.00)	
Age (years)	58 (52–65)	56 (50–62)	58 (52–65)	60 (54–67)	62 (56–70)	<0.001
Sex (male)	4,572 (46.55)	2,089 (46.82)	409 (52.10)	1,629 (45.35)	445 (45.32)	0.006
Living place (urban)	3,512 (35.76)	1,466 (32.86)	281 (35.80)	1,383 (38.50)	382 (38.90)	<0.001
**Education**
Less than lower secondary	8,821 (89.83)	3,970 (88.97)	710 (90.45)	3,247 (90.40)	894 (91.13)	0.157
Upper secondary and vocational training	857 (8.73)	433 (9.70)	69 (8.79)	284 (7.91)	70 (7.24)	
Tertiary	142 (1.45)	59 (0.60)	6 (0.06)	61 (1.70)	16 (1.63)	
**Annual per-capita income**
Poverty	3,213 (37.97)	1,368 (35.68)	249 (37.22)	1,246 (40.21)	350 (40.75)	<0.001
Low income	2,877 (34.00)	1,396 (36.41)	223 (33.33)	994 (32.07)	282 (32.83)	
High income	2,371 (28.02)	1,070 (27.91)	197 (29.45)	859 (32.07)	227 (26.43)	
Basic ADL (score = 0)	8,115 (83.37)	3,803 (86.12)	650 (83.55)	2,914 (81.72)	748 (76.80)	<0.001
Instrumental ADL (score = 0)	7,730 (78.93)	3,656 (82.10)	612 (78.16)	2,762 (77.17)	700 (71.65)	<0.001
Marital status (married)	8,593 (87.50)	4,065 (91.10)	683 (87.01)	3,038 (84.58)	807 (82.18)	<0.001
Smoking	3,823 (38.94)	1,731 (38.79)	357 (45.54)	1,334 (37.15)	401 (40.88)	<0.001
Drinking	3,803 (38.75)	1,704 (38.21)	316 (40.31)	1,407 (39.19)	376 (38.33)	0.635
BMI (kg/m^2^)	23.1 (20.8–25.8)	22.4 (20.4–24.7)	22.6 (20.1–25.4)	24.0 (21.5–26.6)	27.8 (22.0–28.1)	0.028
Diabetes	657 (6.74)	172 (3.88)	44 (5.65)	326 (9.16)	115 (11.82)	<0.001
Dyslipidemia	993 (10.29)	245 (5.57)	37 (4.80)	530 (15.04)	181 (18.87)	<0.001
Heart disease	1,289 (13.17)	387 (8.70)	77 (9.85)	632 (17.64)	193 (19.75)	<0.001
Cancer	107 (1.09)	44 (0.99)	18 (2.30)	33 (0.92)	12 (1.23)	0.007
Lung disease	1,074 (10.97)	432 (9.72)	114 (14.62)	394 (10.98)	134 (13.69)	<0.001
Liver disease	372 (3.81)	160 (3.60)	29 (3.72)	142 (3.98)	41 (4.19)	0.753
Kidney disease	638 (6.52)	282 (6.41)	42 (5.38)	233 (6.52)	78 (7.97)	0.163
Asthma	478 (4.88)	176 (3.95)	67 (8.58)	175 (4.89)	60 (6.13)	<0.001
Arthritis	3,592 (36.64)	1,574 (35.33)	274 (34.90)	1,343 (37.46)	404 (41.00)	0.004
Digestive disease	2,330 (23.76)	1,179 (26.47)	179 (22.86)	21.54 (21.54)	199 (20.31)	<0.001
Memory-related disease	165 (1.68)	55 (1.24)	7 (0.90)	75 (2.09)	28 (2.85)	<0.001
Psychosis-related disease	137 (1.40)	64 (1.44)	8 (1.02)	56 (1.56)	9 (0.92)	0.361

^a^Group 1 is a non-high c-reactive protein (HCRP) and non-hypertension.

^b^Group 2 is HCRP and non-hypertension.

^c^Group 3 is non-HCRP and hypertension.

^d^Group 4 is HCRP and hypertension.

^e^Pearson chi-square test or Kruskal–Wallis test or analysis of variance in multiple groups. Values were presented as n (%), median (25th−75th percentile), mean ± SD.

### Individuals of new-onset stroke risks in the different model

**Figure 2:**
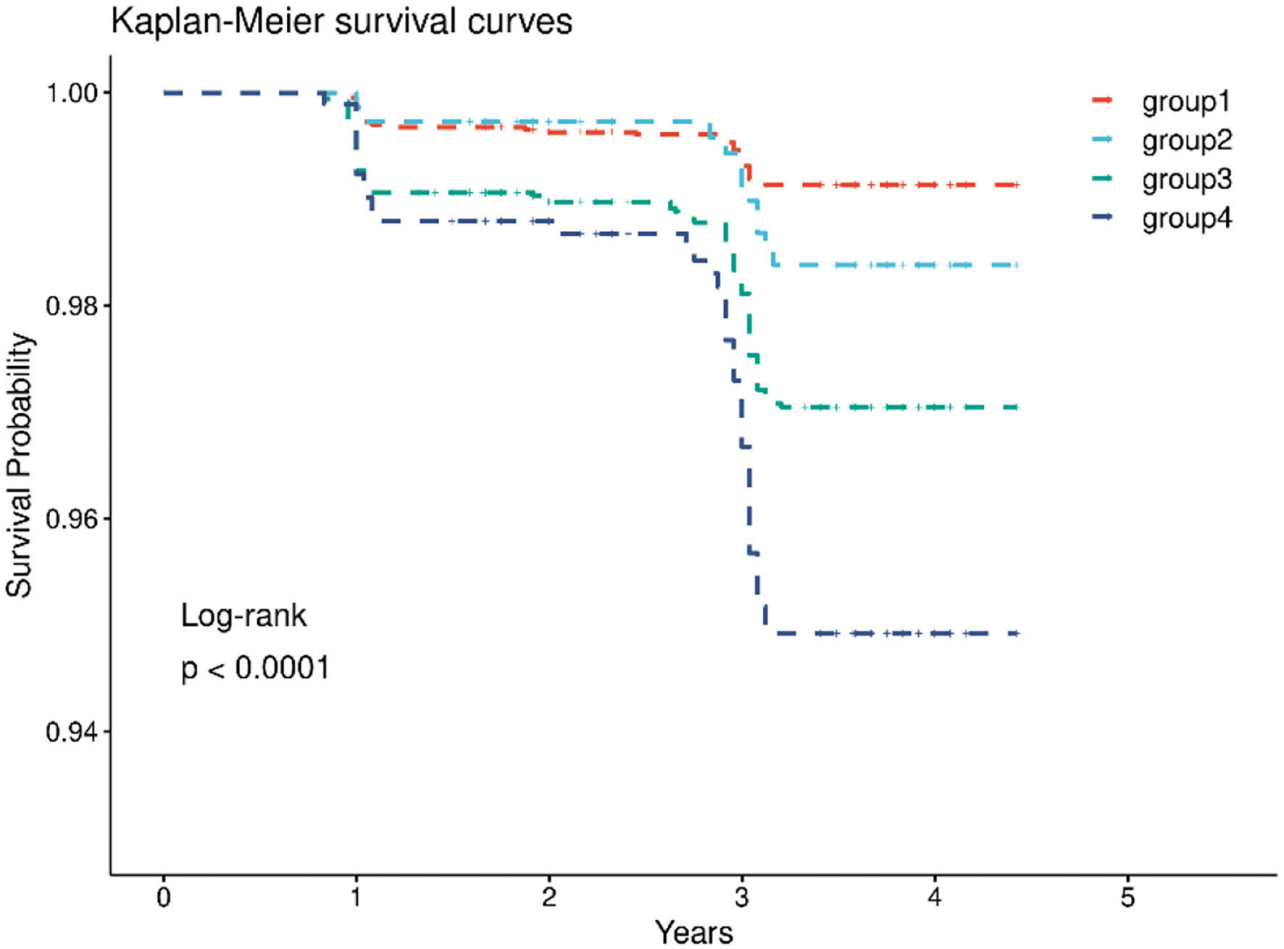
Kaplan–Meier survival curves for the stroke in four groups in CHARLS.

In the cohort, 4,462 (45.4%) had both normal CRP levels and non-hypertension, 785 (8.0%) had elevated-CRP levels alone, 3,592 (36.6%) had hypertension alone, and 982 (10.0%) had both the two conditions. After the 4-year follow-up, the number of strokes in the four groups were 36 (0.81%), 11 (1.40%), 93 (2.64%), and 42 (4.28%) (Table 2), respectively. The Kaplan-Meier plot of stroke according to the elevated-CRP levels and hypertension showed the cumulative incidence rates of new-onset stroke. The survival distributions of the four groups are significantly different (Log-rank test *P* < 0.001); individuals with elevated-CRP levels and hypertension (group 4) possessed a higher risk of new-onset stroke compared to those in the other groups (Figure 2). In the unadjusted model, compared with those in group 1, individuals in group 3 (HR = 3.43, 95% CI: 2.34–5.03) and group 4 (HR = 5.87, 95% CI: 3.76–9.16) had significantly higher risks of new-onset stroke after adjusting for age, sex, annual per-capita income, living place, and education level (model 1). The participants in group 2 (HR = 2.03, 95% CI: 1.01–4.05), group 3 (HR = 3.23, 95% CI: 2.11–4.93), and group 4 (HR = 5.70, 95% CI: 3.51–9.25) had a significantly higher risk of developing stroke. Following further adjustments for smoking status, drinking status, BMI, basic ADL, and instrumental ADL (model 2), [group 2 (HR = 1.79, 95% CI: 0.87–3.68), group 3 (HR = 2.98, 95% CI: 1.91–4.65), and group 4 (HR = 5.34, 95% CI: 3.20–8.92)]. After adjustment for various confounders (model 3), the close associations persisted [group 2 (HR = 1.86, 95% CI: 0.90–3.85), group 3 (HR = 2.70, 95% CI: 1.71–4.28), group 4 (HR = 4.80, 95% CI: 2.83–8.12)]. There was no significant association between elevated-CRP levels and non-hypertension on the risk of new-onset stroke in the unadjusted model, model 2, and model 3 (Table 2). In addition, compared to those with normal CRP levels, individuals with elevated-CRP levels had significantly higher risks of new-onset stroke in all models (Supplementary Table S2). Furthermore, those with hypertension had significantly higher risks of new-onset stroke in all models, compared to those without hypertension (Supplementary Table S3).

**Table 2:** The joint association of elevated-CRP levels and hypertension with new-onset stroke in CHARLS.

**New-onset stroke**	**Group 1**	**Group 2**	**Group 3**	**Group 4**
Cases [*n* (%)]	36/4,462(0.81)	11/785 (1.40)	95/3,592 (2.64)	42/982 (4.28)
Unadjusted	1 (ref)	1.83 (0.93–3.60)	3.43 (2.34–5.03)	5.87 (3.76–9.16)
Model 1^a^	1 (ref)	2.03 (1.01–4.05)	3.23 (2.11–4.93)	5.70 (3.51–9.25)
Model 2^b^	1 (ref)	1.79 (0.87–3.68)	2.98 (1.91–4.65)	5.34 (3.20–8.92)
Model 3^c^	1 (ref)	1.86 (0.90–3.85)	2.70 (1.71–4.28)	4.80 (2.83–8.12)

Group 1 is normal CRP levels and non-hypertension; Group 2 is elevated-CRP levels and non-hypertension; Group 3 is normal.

CRP levels and hypertension; Group 4 is elevated-CRP levels and hypertension. Values were presented as hazard ratios (95% confidence interval).

^a^Model 1: adjusted for age, sex, annual per-capita income, living place, and education level.

^b^Model 2: model 1 with smoking status, drinking status, BMI, basic ADL, and instrumental ADL.

^c^Model 3: model 2 with dyslipidemia, diabetes/high blood sugar, heart problems, cancer, chronic lung disease, memory-related disease, kidney disease, liver disease, arthritis, digestive disease, asthma, and psychiatric problems.

### Sensitivity, subgroup, and the prediction ability analysis

**Figure 3:**
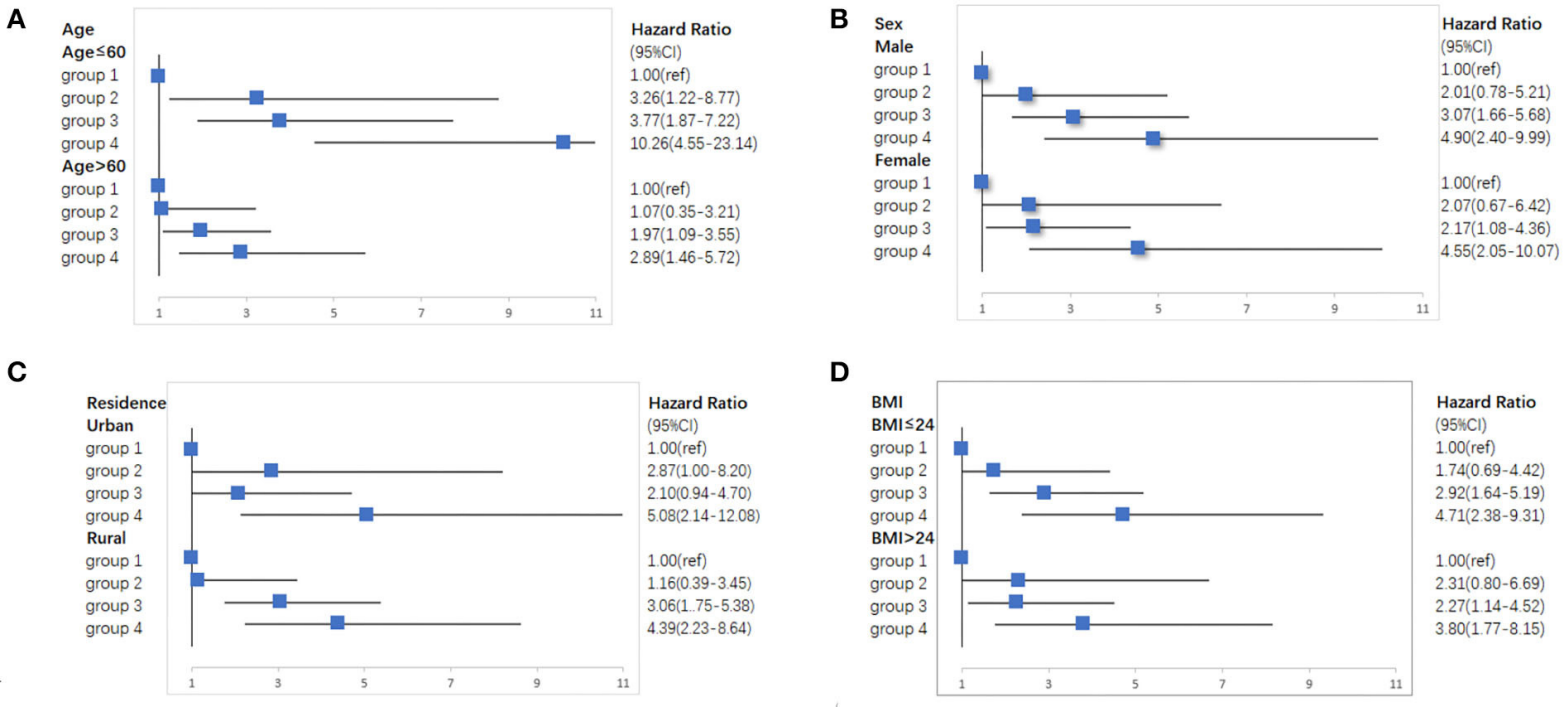
Subgroup analyses of the association of elevated-CRP levels and hypertension with new-onset stroke in CHARLS. **(A)** Subgroup analyses according to age. **(B)** Subgroup analyses according to sex. **(C)** Subgroup analyses according to residence. **(D)** Subgroup analyses according to BMI.

We conducted a series of sensitivity analyses, there was a robust combined effect of elevated-CRP levels and hypertension on new-onset stroke (Supplementary Tables S4–S8). The subgroup analyses demonstrated that individuals with elevated-CRP levels and hypertension have the highest risk of new-onset stroke after adjusting for demographic characteristics, lifestyle factors, and chronic diseases; the risk was especially high in individuals <60 years old (HR = 10.26, 95% CI: 4.55–23.14), men (HR = 4.90, 95% CI: 2.40–9.99), those who lived in the urban areas (HR = 5.08, 95% CI: 2.14–12.08), and those with BMI under 24 kg/m^2^(HR = 4.71, 95% CI: 2.38–9.31). This joint effect remains in the specific analysis (Figure 3).

Compared with the conventional model (AUC = 0.7388), after elevated-CRP levels and hypertension are added to the conventional model, the discrimination and risk reclassification ability can be significantly improved. The value of AUC was 0.7636, the value of NRI was 0.3499 (*P* < 0.0001), and the value of IDI was 0.0052 (*P* = 0.0026) for those with elevated-CRP levels and hypertension (Supplementary Table S9).

## Discussion

In a nationwide prospective cohort study, we found that the combined elevated-CRP levels and hypertension conferred a higher risk for future stroke than each component individually among the middle-aged and geriatric Chinese population, and the combined effect was independent of demographic characteristics, lifestyle factors, chronic diseases, and medication history.

Hypertension is one of the strongest risk factors for stroke worldwide (23, 24). Recent evidence focuses on the relationship between elevated-CRP levels and ischemic stroke (25), fatal stroke (26), non-fatal stroke (27), and transient ischemic attack (28). However, a few studies on the relationship between CRP and hemorrhagic stroke indicate that elevated-CRP levels are not associated with hemorrhagic stroke (7). In addition, there were inconsistent results in overall stroke (27, 29). Based on these studies, we conducted our study to assess the combined effect of elevated-CRP levels and hypertension on the overall risk of stroke in the middle-aged and geriatric Chinese population.

Our study is one of the studies that support the evidence that the combined effect of elevated-CRP levels and hypertension can potentially increase the risk of stroke in the middle-aged and geriatric Chinese population. Several explanations have been put forward to explain the possible underlying mechanisms. Hypertension may promote inflammation of the blood vessels by increasing mechanical stress on arterial walls (30) and normal CRP levels could be prevented against hypertension in animal models and humans (31, 32). There are many mechanisms by which CRP levels may increase BP by regulating many molecules released from endothelial cells. The co-occurrence of both may mutually promote, finally, increasing the risk of stroke by elevating blood pressure. Previous studies have shown that the burden due to stroke can be attributed to modifiable atherosclerotic risk factors. CRP may predict the burden of atherosclerosis, since it is a systemic marker of inflammation, and is also aggravated by high blood pressure (33). The latter is well-known as the pathological finding of cerebrovascular diseases (34). A study showing elevated-CRP levels was associated with cerebral micro-bleeding in both lobar and deep locations (35). The elevated-CRP levels may promote thrombotic events by inducing monocytes to express tissue factors for IS (36), additionally, the small arteries seem prone to hypertension-induced vascular injury (37). Therefore, we believe that the co-occurrence of CRP levels elevation and hypertension can accelerate the progression of atherosclerosis and cerebral vascular injury than the separation of both performed. However, further MRI evidence is needed to verify this in future studies. In addition to this, hypertension can independently trigger the different stages of atherogenesis, which rely on oxidative stress, endothelial dysfunction, and inflammation (33). Therefore, hypertension is a powerful trigger for vascular inflammation, and CRP provides a synergistic effect, leading to an increase in the risk of stroke among individuals.

The current study has shown that CRP concentration is as consistent within individuals as the total cholesterol concentration and systolic blood pressure over several years (25). This stable existence of CRP concentration rather than transient makes it possible to measure accurate value. Previous studies showed that there were inconsistent results in overall stroke (27, 29). The subgroup analyses proved that individuals with elevated-CRP levels and hypertension have the risk of new cases of stroke in all subgroups. Other groups of populations have a higher risk of stroke: those who were male, aged <60 years, lived in the urban, or with BMI under 24 kg/m^2^. Population-based studies have demonstrated that age-adjusted stroke incidence rates were higher in men than in women worldwide (10). It has been reported that exposure to endogenous estrogen protects premenopausal women from stroke (38). Furthermore, people <60 years and who lived in urban areas were more likely to experience psychological distress as a result of work, poor sleep quality, and specific dietary factors, which could also be associated with a greater risk of stroke (39–41). Those overweight or with obesity may have a protective effect on recurrent stroke events among patients with stroke (42); it can partly explain the low relative risk of stroke in individuals with elevated-CRP levels, hypertension, and obesity. Thus, our study will contribute toward better prevention of stroke.

Over the past several decades, stroke has become a leading cause of mortality and disability worldwide and there are substantial economic costs for post-stroke care, and 13.8% of participants with hypertension have elevated-CRP levels in CHARLS (20). However, previous studies were limited to using elevated-CRP levels as a predictor of IS, not overall stroke. The association of high CRP and hypertension with stroke sub-type (hemorrhage and infarction) will be further investigated in future studies. Our study demonstrates that screening and monitoring individuals with high CRP levels and hypertension using easy-access tools can be effective in the prevention of stroke. Several non-drug and pharmacological blood-pressure-lowering therapies can reduce CRP levels, unfortunately, the proportion of patients who regularly take antihypertensive drugs is 30.1% (12). It may result in limited clinical applicability. In community screening, clinicians can require careful monitoring of C-reactive protein and blood pressure to screen high-risk populations and provide accurate prevention and control, such as improving their lifestyles, or to comply with drug therapies.

However, this study had certain limitations. First, the cut-off value of the CRP concentrations is a key point, but the elevated-CRP levels did not have a single criterion because the cut-off value used in this present study is often used in other studies (4, 43, 44). Hypertension in this study was defined as a single entity including both treated and untreated patients. We conducted a sensitivity analysis to investigate the association of elevated-CRP levels and untreated hypertension with new-onset stroke after excluding those with antihypertensive treatment (Supplementary Table S8); there was a robust combined effect of elevated-CRP levels and hypertension on stroke. Second, information on stroke is generally self-reported, which increases the risk of information bias, but good reliability has been proven between self-reported stroke and stroke diagnosed by a doctor (45, 46). Moreover, two sensitivity analysis of different definitions of hypertension was also performed (Supplementary Tables S4, S8). Third, some individuals were excluded due to incomplete information on CRP concentration and hypertension on the baseline. This may induce a selection bias. Most of these characteristics were significantly different (*P* < 0.05) due to the large sample size, but the differences were not large from a clinical perspective (Supplementary Table S1). Moreover, we adjusted for these potential influencing factors in our multivariate analysis. Fourth, considering that the differences in the AUC between adding elevated-CRP levels and hypertension to the conventional model were relatively small. It may result in limited clinical applicability. Finally, there were some missing values for various covariates, but the proportion was <5%, which may not have prevented the discovery of the associations in the multivariable analysis.

In conclusion, our study results showed that the combined effect of elevated-CRP levels and hypertension can increase the risk of stroke among the middle-aged and geriatric Chinese population, and corresponding interventions may be worthwhile for stroke prevention. Therefore, future studies are needed to evaluate the predictive ability of elevated-CRP levels and hypertension on new-onset stroke with a longer duration of follow-up; furthermore, the degree of changes in atherosclerosis due to elevated-CRP levels and hypertension needs to be assessed to verify our hypothesis.

## Data availability statement

The datasets generated and/or analyzed during the current study are available in the CHARLS repository, http://charls.pku.edu.cn.

## Ethics statement

The current study is a secondary analysis of the de-identified China Health and Retirement Longitudinal Study (CHARLS) public data. The original CHARLS was approved by the Biomedical Ethics Review Committee of Peking University (IRB00001052–11015). Additionally, written informed consent has been obtained from all participants. All methods were performed according to the Declaration of Helsinki.

## Author contributions

YS and SL designed the research. XC wrote the main manuscript. XC and SL performed the data analysis. WH, NS, and JC provided all tables and figures. All authors contributed to the interpretations of the findings and reviewed the manuscript.

## Funding

This study was supported by the National Natural Science Foundation of China under Grant Number 81973143 and the Priority Academic Program Development of Jiangsu Higher Education Institutions (PAPD).

## Conflict of interest

The authors declare that the research was conducted in the absence of any commercial or financial relationships that could be construed as a potential conflict of interest. The reviewer MZ declared a shared affiliation, though no collaboration, with the authors XC, SL, JC, WH, NS, and YS to the handling editor at the time of review.

## Publisher's note

All claims expressed in this article are solely those of the authors and do not necessarily represent those of their affiliated organizations, or those of the publisher, the editors and the reviewers. Any product that may be evaluated in this article, or claim that may be made by its manufacturer, is not guaranteed or endorsed by the publisher.

## Abbreviations

CHARLS: China Health and Retirement Longitudinal Study
AUC: area under the curve
NRI: net reclassification index
IDI: integrated discrimination improvement
CRP: C-reactive protein
DALYs: Disability-adjusted life years
SBP: Systolic blood pressure
DBP: Diastolic blood pressure
ADL: Activities of Daily Living Scale
API: Annual per-capita income.

## References

[1] Rajsic S Gothe H Borba HH Sroczynski G Vujicic J Toell T. Economic burden of stroke: a systematic review on post-stroke care. Eur J Health Econ. (2019) 20:107–34. 10.1007/s10198-018-0984-0 29909569

[2] Johnson CO Nguyen M Roth GA Nichols E Alam T Abate D. Global, regional, and national burden of stroke, 1990–2016: a systematic analysis for the Global Burden of Disease Study 2016. Lancet Neurol. (2019) 18:439–58. English. 10.1016/S1474-4422(19)30034-1 30871944 PMC6494974

[3] Wu S Wu B Liu M Chen Z Wang W Anderson CS. Stroke in China: advances and challenges in epidemiology, prevention, and management. Lancet Neurol. (2019) 18:394–405. eng. 10.1016/S1474-4422(18)30500-3 30878104

[4] Patgiri D Pathak MS Sharma P Kutum T Mattack N. Serum hsCRP: a novel marker for prediction of cerebrovascular accidents (stroke). J Clin Diagn Res. (2014) 8:Cc08–11. eng. 10.7860/JCDR/2014/10386.5302 25653940 PMC4316246

[5] Black S Kushner I Samols D. C-reactive protein. J Biol Chem. (2004) 279:48487–90. 10.1074/jbc.R400025200 15337754

[6] Tzoulaki I Murray GD Lee AJ Rumley A Lowe GD Fowkes FG. C-reactive protein, interleukin-6, and soluble adhesion molecules as predictors of progressive peripheral atherosclerosis in the general population: Edinburgh Artery Study. Circulation. (2005) 112:976–83. 10.1161/CIRCULATIONAHA.104.513085 16087797

[7] Zhou Y Han W Gong D Man C Fan Y. Hs-CRP in stroke: a meta-analysis. Clin Chim Acta. (2016) 453:21–7. 10.1016/j.cca.2015.11.027 26633855

[8] Forouzanfar MH Liu P Roth GA Ng M Biryukov S Marczak L. Global burden of hypertension and systolic blood pressure of at least 110 to 115 mm Hg, 1990–2015. JAMA. (2017) 317:165–82. 10.1001/jama.2016.19043 28097354

[9] Zhou M Wang H Zeng X Yin P Zhu J Chen W. Mortality, morbidity, and risk factors in China and its provinces, 1990–2017: a systematic analysis for the Global Burden of Disease Study 2017. Lancet. (2019) 394:1145–58. 10.1016/S0140-6736(19)30427-1 31248666 PMC6891889

[10] Virani SS Alonso A Benjamin EJ Bittencourt MS Callaway CW Carson AP. Heart disease and stroke statistics-2020 update: a report from the American Heart Association. Circulation. (2020) 141:e139–596. 10.1161/CIR.0000000000000757 31992061

[11] Wang W Jiang B Sun H Ru X Sun D Wang L. Prevalence, incidence, and mortality of stroke in China: results from a nationwide population-based survey of 480 687 adults. Circulation. (2017) 135:759–71. 10.1161/CIRCULATIONAHA.116.025250 28052979

[12] Lu J Lu Y Wang X Li X Linderman GC Wu C. Prevalence, awareness, treatment, and control of hypertension in China: data from 1·7 million adults in a population-based screening study (China PEACE Million Persons Project). Lancet. (2017) 390:2549–58. 10.1016/S0140-6736(17)32478-9 29102084

[13] Kim JS Caplan LR. Clinical stroke syndromes. Front Neurol Neurosci. (2016) 40:72–92. 10.1159/000448303 27960164

[14] Zhao Y Hu Y Smith JP Strauss J Yang G. Cohort profile: the China Health and Retirement Longitudinal Study (CHARLS). Int J Epidemiol. (2014) 43:61–8. 10.1093/ije/dys203 23243115 PMC3937970

[15] Liu S Qiao Y Zhang Y Wu Y Ke C Shen Y. Combined effect of high depressive symptom burden and hypertension on new-onset stroke: evidence from a nationwide prospective cohort study. J Hypertens. (2021) 39:70–6. 10.1097/HJH.0000000000002599 32740408

[16] Jiménez MC Rexrode KM Glynn RJ Ridker PM Gaziano JM Sesso HD. Association between high-sensitivity c-reactive protein and total stroke by hypertensive status among men. J Am Heart Assoc. (2015) 4:e002073. 10.1161/JAHA.115.002073 26391131 PMC4599494

[17] Joint Committee for Guideline Revision. 2018 Chinese guidelines for prevention and treatment of hypertension-a report of the revision committee of Chinese guidelines for prevention and treatment of hypertension. J Geriatr Cardiol. (2019) 16:182–241. 10.11909/j.issn.1671-5411.2019.03.014 31080465 PMC6500570

[18] Lin L Wang HH Lu C Chen W Guo VY. Adverse childhood experiences and subsequent chronic diseases among middle-aged or older adults in China and associations with demographic and socioeconomic characteristics. JAMA Netw Open. (2021) 4:e2130143. 10.1001/jamanetworkopen.2021.30143 34694390 PMC8546496

[19] Jiang Y Zheng H Zhao T. Socioeconomic status and morbidity rate inequality in China: based on NHSS and CHARLS data. Int J Environ Res Public Health. (2019) 16:215. 10.3390/ijerph16020215 30646540 PMC6351904

[20] Qin T Liu W Yin M Shu C Yan M Zhang J. Body mass index moderates the relationship between C-reactive protein and depressive symptoms: evidence from the China Health and Retirement Longitudinal Study. Sci Rep. (2017) 7:39940. 10.1038/srep39940 28128231 PMC5269588

[21] Wu C. The mediating and moderating effects of depressive symptoms on the prospective association between cognitive function and activities of daily living disability in older adults. Arch Gerontol Geriatr. (2021) 96:104480. 10.1016/j.archger.2021.104480 34274875

[22] Whelton PK Carey RM Aronow WS Casey DE Jr Collins KJ Dennison Himmelfarb C. 2017. ACC/AHA/AAPA/ABC/ACPM/AGS/APhA/ASH/ASPC/NMA/PCNA guideline for the prevention, detection, evaluation, and management of high blood pressure in adults: a report of the American College of Cardiology/American Heart Association Task Force on Clinical Practice Guidelines. J Am Coll Cardiol. (2018) 71:e127–248. 10.1016/j.jacc.2017.11.006 29146535

[23] Wang J Wen X Li W Li X Wang Y Lu W. Risk factors for stroke in the chinese population: a systematic review and meta-analysis. J Stroke Cerebrovasc Dis. (2017) 26:509–17. 10.1016/j.jstrokecerebrovasdis.2016.12.002 28041900

[24] Ettehad D Emdin CA Kiran A Anderson SG Callender T Emberson J. Blood pressure lowering for prevention of cardiovascular disease and death: a systematic review and meta-analysis. Lancet. (2016) 387:957–67. 10.1016/s0140-6736(15)01225-8 26724178

[25] Kaptoge S Di Angelantonio E Lowe G Pepys MB Thompson SG Collins R. C-reactive protein concentration and risk of coronary heart disease, stroke, and mortality: an individual participant meta-analysis. Lancet. (2010) 375:132–40. 10.1016/S0140-6736(09)61717-7 20031199 PMC3162187

[26] Gussekloo J Schaap MC Frölich M Blauw GJ Westendorp RG. C-reactive protein is a strong but nonspecific risk factor of fatal stroke in elderly persons. Arterioscler Thromb Vasc Biol. (2000) 20:1047–51. 10.1161/01.ATV.20.4.1047 10764671

[27] Liu Y Wang J Zhang L Wang C Wu J Zhou Y. Relationship between C-reactive protein and stroke: a large prospective community based study. PLoS ONE. (2014) 9:e107017. 10.1371/journal.pone.0107017 25191699 PMC4156395

[28] Rost NS Wolf PA Kase CS Kelly-Hayes M Silbershatz H Massaro JM. Plasma concentration of C-reactive protein and risk of ischemic stroke and transient ischemic attack: the Framingham study. Stroke. (2001) 32:2575–9. 10.1161/hs1101.098151 11692019

[29] Chei CL Yamagishi K Kitamura A Kiyama M Imano H Ohira T. C-reactive protein levels and risk of stroke and its subtype in Japanese: the Circulatory Risk in Communities Study (CIRCS). Atherosclerosis. (2011) 217:187–93. 10.1016/j.atherosclerosis.2011.03.001 21444086

[30] Hage FG. C-reactive protein and hypertension. J Hum Hypertens. (2014) 28:410–5. 10.1038/jhh.2013.111 24226100

[31] Ratto E Leoncini G Viazzi F Falqui V Parodi A Conti N. C-reactive protein and target organ damage in untreated patients with primary hypertension. J Am Soc Hypertens. (2007) 1:407–13. 10.1016/j.jash.2007.09.003 20409873

[32] Jialal I Devaraj S Siegel D. CRP induces hypertension in animal models: homo sapiens says no. Hypertens Res. (2011) 34:801–2. 10.1038/hr.2011.59 21593738

[33] Hurtubise J McLellan K Durr K Onasanya O Nwabuko D Ndisang JF. The different facets of dyslipidemia and hypertension in atherosclerosis. Curr Atheroscler Rep. (2016) 18:82. 10.1007/s11883-016-0632-z 27822682

[34] Banerjee C Chimowitz MI. Stroke caused by atherosclerosis of the major intracranial arteries. Circ Res. (2017) 120:502–13. 10.1161/CIRCRESAHA.116.308441 28154100 PMC5312775

[35] Mitaki S Nagai A Oguro H Yamaguchi S. C-reactive protein levels are associated with cerebral small vessel-related lesions. Acta Neurol Scand. (2016) 133:68–74. 10.1111/ane.12440 25974422

[36] Cermak J Key NS Bach RR Balla J Jacob HS Vercellotti GM. C-reactive protein induces human peripheral blood monocytes to synthesize tissue factor. Blood. (1993) 82:513–20. 10.1182/blood.V82.2.513.bloodjournal822513 8329706

[37] Diaz-Otero JM Fisher C Downs K Moss ME Jaffe IZ Jackson WF. Endothelial mineralocorticoid receptor mediates parenchymal arteriole and posterior cerebral artery remodeling during angiotensin ii-induced hypertension. Hypertension. (2017) 70:1113–21. 10.1161/HYPERTENSIONAHA.117.09598 28974571 PMC5680120

[38] Bushnell C McCullough LD Awad IA Chireau MV Fedder WN Furie KL. Guidelines for the prevention of stroke in women: a statement for healthcare professionals from the American Heart Association/American Stroke Association. Stroke. (2014) 45:1545–88. 10.1161/01.str.0000442009.06663.48 24503673 PMC10152977

[39] Jackson CA Sudlow CLM Mishra GD. Psychological distress and risk of myocardial infarction and stroke in the 45 and up study. Circ Cardiovasc Qual Outcomes. (2018) 11:e004500. 10.1161/circoutcomes.117.004500 30354546

[40] Zhou L Yu K Yang L Wang H Xiao Y Qiu G. Sleep duration, midday napping, and sleep quality and incident stroke: the Dongfeng-Tongji cohort. Neurology. (2020) 94:e345–56. 10.1212/wnl.0000000000008739 31827003

[41] Micha R Peñalvo JL Cudhea F Imamura F Rehm CD Mozaffarian D. Association between dietary factors and mortality from heart disease, stroke, and type 2 diabetes in the United States. JAMA. (2017) 317:912–24. 10.1001/jama.2017.0947 28267855 PMC5852674

[42] Huang K Liu F Han X Huang C Huang J Gu D. Association of BMI with total mortality and recurrent stroke among stroke patients: a meta-analysis of cohort studies. Atherosclerosis. (2016) 253:94–101. 10.1016/j.atherosclerosis.2016.08.042 27596134

[43] DuBrock HM AbouEzzeddine OF Redfield MM. High-sensitivity C-reactive protein in heart failure with preserved ejection fraction. PLoS ONE. (2018) 13:e0201836. 10.1371/journal.pone.0201836 30114262 PMC6095520

[44] Shen Y Zhang Y Xiong S Zhu X Ke C. High-sensitivity C-reactive protein and cystatin C independently and jointly predict all-cause mortality among the middle-aged and elderly Chinese population. Clin Biochem. (2019) 65:7–14. 10.1016/j.clinbiochem.2018.12.012 30592989

[45] Yuan X Liu T Wu L Zou ZY Li C. Validity of self-reported diabetes among middle-aged and older Chinese adults: the China Health and Retirement Longitudinal Study. BMJ Open. (2015) 5:e006633. 10.1136/bmjopen-2014-006633 25872937 PMC4401856

[46] Choe S Lee J Lee J Kang D Lee JK Shin A. Validity of self-reported stroke and myocardial infarction in Korea: the Health Examinees (HEXA) study. J Prev Med Public Health. (2019) 52:377–83. 10.3961/jpmph.19.089 31795614 PMC6893227

