# Supplementary material for: Joint effect of elevated-c-reactive protein level and hypertension on new-onset stroke: A nationwide prospective cohort study of CHARLS
Source: Front Public Health. 2022 Oct 3;10:919506. doi: 10.3389/fpubh.2022.919506 (PMC9573958; doi:10.3389/fpubh.2022.919506)
Supplement: Supplementary file 1 [file Data_Sheet_1.docx]

Joint effect of elevated-C-reactive protein level and hypertension on new-onset stroke: a nationwide prospective cohort study of CHARLS (Supplement materials)

Table S1. Baseline characteristics of participants and non-participants in CHARLS

| Variables(%) | All | non-participants | participants | *P* value^a^ |
| --- | --- | --- | --- | --- |
| N | 17,224 | 7,403(42.98) | 9,821(57.02) |  |
| Age(years) | 58(51-65) | 57(50-65) | 58(52-65) | <0.001 |
| Sex(Male) | 8,397(48.75) | 3,825(51.67) | 4,572(46.55) | <0.001 |
| Living place (Urban) | 6,967(40.45) | 3,455(46.67) | 3,512(35.76) | <0.001 |
| Education |  |  |  |  |
| Less than lower secondary | 15,032(87.32) | 6,211(84.00) | 8,821(89.83) | <0.001 |
| Upper secondary & vocational training | 1,796(10.43) | 939(12.70) | 857(8.73) |  |
| Tertiary | 386(2.24) | 244(3.30) | 142(1.45) |  |
| Annual per-capita income |  |  |  |  |
| Poverty | 5,106(35.40) | 1,893(31.76) | 3,213(37.97) | <0.001 |
| Low income | 4,812(33.37) | 1,917(32.16) | 2,895(34.22) |  |
| High income | 4,504(31.23) | 2,151(36.08) | 2,353(27.81) |  |
| Basic ADL (Score=0) | 14,040(83.05) | 5,925(82.61) | 8,115(83.37) | 0.196 |
| Instrumental ADL (Score=0) | 13,337(78.27) | 5,607(77.37) | 7,730(78.93) | 0.014 |
| Marital status (Married) | 14,970(86.94) | 6,377(86.21) | 8,593(87.50) | 0.013 |
| Smoking | 6,867(40.16) | 3,044(41.81) | 3,823(38.94) | <0.001 |
| Drinking | 6,733(39.42) | 2,930(40.32) | 3,803(38.75) | 0.037 |
| BMI (kg/m^2^) | 23.5±3.95 | 23.3±3.99 | 23.5±3.93 | 0.013 |
| Diabetes | 1,099(6.48) | 442(6.13) | 657(6.74) | 0.111 |
| Dyslipidemia | 1,674(9.99) | 681(9.57) | 993(10.29) | 0.124 |
| Heart disease | 2,186(12.85) | 897(12.42) | 1,289(13.17) | 0.153 |
| Cancer | 184(1.08) | 77(1.06) | 107(1.09) | 0.854 |
| Lung disease | 1,810(10.62) | 736(10.16) | 1,074(10.97) | 0.089 |
| Liver disease | 641(3.77) | 269(3.73) | 372(3.81) | 0.792 |
| Kidney disease | 1,041(6.13) | 403(5.59) | 638(6.52) | 0.012 |
| Asthma | 817(4.80) | 339(4.68) | 478(4.88) | 0.540 |
| Arthritis | 5,873(34.42) | 2,281(31.41) | 3,592(36.64) | <0.001 |
| Digestive disease | 3,834(22.48) | 1,504(20.74) | 2,330(23.76) | <0.001 |
| Memory-related disease | 366(2.15) | 201(2.77) | 165(1.68) | <0.001 |
| Psychosis-related disease | 268(1.57) | 131(1.81) | 137(1.40) | 0.033 |

Table S2. Association of elevated-CRP levels and new-onset stroke in CAHRLS (2011-2015)

| New-onset stroke | Group 1 | Group 2 |
| --- | --- | --- |
| Cases [n (%)] | 131/8054(1.63) | 53/1767(3.00) |
| Unadjusted | 1(ref) | 1.96(1.42-2.69) |
| Model 1^a^ | 1(ref) | 1.97(1.41-2.76) |
| Model 2^b^ | 1(ref) | 1.90(1.33-2.70) |
| Model 3^c^ | 1(ref) | 1.88(1.31-2.70) |

Group 1 is normal CRP levels; Group 2 is elevated-CRP levels. Values were presented as hazard ratios (95% confidence interval).
^a^ Model 1: adjusted for age, sex, annual per-capita income, living place, and education level.
^b^ Model 2: model 1 with smoking status, drinking status, BMI, basic ADL, and instrumental ADL.
^c^ Model 3: model 2 with dyslipidemia, diabetes/high blood sugar, heart problems, cancer, chronic lung disease, memory-related disease, kidney disease, liver disease, arthritis, digestive disease, asthma, psychiatric problems.

Table S3. Association of hypertension and new-onset stroke in CAHRLS (2011-2015)

| New-onset stroke | Group 1 | Group 2 |
| --- | --- | --- |
| Cases [n (%)] | 47/5247(0.90) | 137/4574(3.00) |
| Unadjusted | 1(ref) | 3.51(2.52-4.89) |
| Model 1^a^ | 1(ref) | 3.21(2.24-4.61) |
| Model 2^b^ | 1(ref) | 3.05(2.08-4.47) |
| Model 3^c^ | 1(ref) | 2.73(1.84-4.05) |

Group 1 is non-hypertension; Group 2 is hypertension. Values were presented as hazard ratios (95% confidence interval).
^a^ Model 1: adjusted for age, sex, annual per-capita income, living place, and education level.
^b^ Model 2: model 1 with smoking status, drinking status, BMI, basic ADL, and instrumental ADL.
^c^ Model 3: model 2 with dyslipidemia, diabetes/high blood sugar, heart problems, cancer, chronic lung disease, memory-related disease, kidney disease, liver disease, arthritis, digestive disease, asthma, psychiatric problems.

Table S4. Sensitivity analysis considering different definition of hypertension for association of elevated-CRP levels and hypertension^*^ with new-onset stroke in CAHRLS (2011-2015)

| New-onset stroke | Group 1 | Group 2 | Group 3 | Group 4 |
| --- | --- | --- | --- | --- |
| Cases [n (%)] | 24/3303(0.73) | 5/546(0.92) | 107/4759(2.25) | 49/1226(4.00) |
| Unadjusted | 1(ref) | 1.33(0.51-3.49) | 3.21(2.06-4.99) | 6.03(3.70-9.83) |
| Model 1^a^ | 1(ref) | 1.40(0.53-3.71) | 2.78(1.72-4.48) | 5.48(3.24-9.25) |
| Model 2^b^ | 1(ref) | 1.38(0.52-3.67) | 2.58(1.56-4.25) | 5.02(2.89-8.73) |
| Model 3^c^ | 1(ref) | 1.47(0.54-3.94) | 2.46(1.47-4.12) | 4.60(2.60-8.15) |

^*^ Hypertension was defined as SBP at least 130 mmHg, DBP at least 80 mmHg, a self-reported history of hypertension, with antihypertensive drug,

Group 1 is normal CRP levels and non-hypertension; Group 2 is elevated-CRP levels and non-hypertension; Group 3 is normal CRP levels and hypertension; Group 4 is elevated-CRP levels and hypertension. Values were presented as hazard ratios (95% confidence interval).
^a^ Model 1: adjusted for age, sex, annual per-capita income, living place, and education level.
^b^ Model 2: model 1 with smoking status, drinking status, BMI, basic ADL, and instrumental ADL.
^c^ Model 3: model 2 with dyslipidemia, diabetes/high blood sugar, heart problems, cancer, chronic lung disease, memory-related disease, kidney disease, liver disease, arthritis, digestive disease, asthma, psychiatric problems.

Table S5. Sensitivity analysis considering individuals developed hypertension during CHARLS 2013 follow-up (n=156) for association of elevated-CRP levels and hypertension^*^ with new-onset stroke in CAHRLS (2011-2015)

| New-onset stroke | Group 1 | Group 2 | Group 3 | Group 4 |
| --- | --- | --- | --- | --- |
| Cases [n (%)] | 31/4336(0.71) | 10/755(1.32) | 100/3718(2.69) | 43/1012(4.25) |
| Unadjusted | 1(ref) | 1.95(0.96-3.98) | 3.92(2.62-5.87) | 6.57(4.14-10.42) |
| Model 1^a^ | 1(ref) | 2.22(1.06-4.63) | 3.86(2.46-6.04) | 6.65(4.00-11.06) |
| Model 2^b^ | 1(ref) | 1.96(0.91-4.23) | 3.64(2.27-5.83) | 6.33(3.70-10.84) |
| Model 3^c^ | 1(ref) | 2.03(0.94-4.41) | 3.36(2.07-5.46) | 5.84(3.37-10.15) |

^*^ 156 individuals developed hypertension during CHARLS 2013 follow-up,

Group 1 is normal CRP levels and non-hypertension; Group 2 is elevated-CRP levels and non-hypertension; Group 3 is normal CRP levels and hypertension; Group 4 is elevated-CRP levels and hypertension. Values were presented as hazard ratios (95% confidence interval).
^a^ Model 1: adjusted for age, sex, annual per-capita income, living place, and education level.
^b^ Model 2: model 1 with smoking status, drinking status, BMI, basic ADL, and instrumental ADL.
^c^ Model 3: model 2 with dyslipidemia, diabetes/high blood sugar, heart problems, cancer, chronic lung disease, memory-related disease, kidney disease, liver disease, arthritis, digestive disease, asthma, psychiatric problems.

Table S6. Sensitivity analysis considering different stage hypertension for association of elevated-CRP levels and hypertension with new-onset stroke in CAHRLS (2011-2015)

| New-onset stroke | Group 1 | Group 2 | Group 3 | Group 4 | Group 5 | Group 6 |
| --- | --- | --- | --- | --- | --- | --- |
| Cases [n (%)] | 31/4336(0.81) | 11/785(1.40) | 93/3422(2.72) | 2/170(1.18) | 40/929(4.31) | 2/53(3.77) |
| Unadjusted | 1(ref) | 1.83(0.93-3.60) | 3.51(2.39-5.16) | 1.62(0.39-6.71) | 5.93(3.77-9.30) | 4.97(1.20-20.64) |
| Model 1^a^ | 1(ref) | 2.03(1.01-4.05) | 3.29(2.15-5.03) | 1.76(0.42-7.38) | 5.75(3.52-9.39) | 4.69(1.11-19.75) |
| Model 2^b^ | 1(ref) | 1.79(0.87-3.68) | 3.03(1.94-4.75) | 1.82(0.43-7.69) | 5.41(3.22-9.09) | 4.36(1.03-18.48) |
| Model 3^c^ | 1(ref) | 1.86(0.90-3.85) | 2.74(1.72-4.34) | 1.99(0.47-8.40) | 4.80(2.81-8.18) | 4.91(1.14-21.08) |

Group 1 is normal CRP levels and non-hypertension; Group 2 is elevated-CRP levels and non-hypertension; Group 3 is normal CRP levels and stage 1 hypertension; Group 4 is normal CRP levels and stage 2-3 hypertension; Group 5 is elevated-CRP levels and stage 1 hypertension Group 6 is elevated-CRP levels and stage 2-3 hypertension. Values were presented as hazard ratios (95% confidence interval).
^a^ Model 1: adjusted for age, sex, annual per-capita income, living place, and education level.
^b^ Model 2: model 1 with smoking status, drinking status, BMI, basic ADL, and instrumental ADL.
^c^ Model 3: model 2 with dyslipidemia, diabetes/high blood sugar, heart problems, cancer, chronic lung disease, memory-related disease, kidney disease, liver disease, arthritis, digestive disease, asthma, psychiatric problems.

Table S7. Sensitivity analysis considering medications history for association of elevated-CRP levels and hypertension with new-onset stroke in CAHRLS (2011-2015)

| New-onset stroke | Group 1 | Group 2 | Group 3 | Group 4 |
| --- | --- | --- | --- | --- |
| Cases [n (%)] | 36/4462(0.81) | 11/785(1.32) | 95/3592(2.64) | 42/982(4.28) |
| Unadjusted | 1(ref) | 1.83(0.93-3.60) | 3.43(2.34-5.03) | 5.87(3.76-9.16) |
| Model 1^a^ | 1(ref) | 2.03(1.01-4.05) | 3.23(2.11-4.93) | 5.70(3.51-9.25) |
| Model 2^b^ | 1(ref) | 1.86(0.90-3.85) | 2.70(1.71-4.28) | 4.80(2.83-8.12) |
| Model 3^c^ | 1(ref) | 1.87(0.90-3.85) | 2.41(1.47-3.97) | 4.22(2.39-7.46) |

Group 1 is normal CRP levels and non-hypertension; Group 2 is elevated-CRP levels and non-hypertension; Group 3 is normal CRP levels and hypertension; Group 4 is elevated-CRP levels and hypertension. Values were presented as hazard ratios (95% confidence interval).
^a^ Model 1: adjusted for age, sex, annual per-capita income, living place, and education level.

^b^ Model 2: model 1 with smoking status, drinking status, BMI, basic ADL, instrumental ADL, dyslipidemia, diabetes/high blood sugar, heart problems, cancer, chronic lung disease, memory-related disease, kidney disease, liver disease, arthritis, digestive disease, asthma, psychiatric problems.
^c^ Model 3: model 2 with medications use for hypertension, diabetes, and dyslipidemia.

Table S8. Sensitivity analysis excluded treated hypertension patients (N=2,806) for association of elevated-CRP levels and hypertension^*^ with new-onset stroke in CAHRLS (2011-2015)

| New-onset stroke | Group 1 | Group 2 | Group 3 | Group 4 |
| --- | --- | --- | --- | --- |
| Cases [n (%)] | 36/4,462(0.81) | 11/785(1.40) | 25/1,437(1.74) | 14/329(4.28) |
| Unadjusted | 1(ref) | 1.83(0.93-3.60) | 2.22(1.33-3.69) | 5.74(3.10-10.64) |
| Model 1^a^ | 1(ref) | 2.03(1.01-4.06) | 2.04(1.16-3.60) | 5.20(2.66-10.17) |
| Model 2^b^ | 1(ref) | 1.70(0.83-3.51) | 1.82(1.01-3.28) | 4.50(2.24-9.03) |
| Model 3^c^ | 1(ref) | 1.86(0.90-3.89) | 1.99(1.09-3.61) | 5.17(2.53-10.56) |

^*^ is sensitivity analysis with excluded treated hypertension patients (N=2,806)

Group 1 is normal CRP levels and non-hypertension; Group 2 is elevated-CRP levels and non-hypertension; Group 3 is normal CRP levels and hypertension; Group 4 is elevated-CRP levels and hypertension. Values were presented as hazard ratios (95% confidence interval).
^a^ Model 1: adjusted for age, sex, annual per-capita income, living place, and education level.
^b^ Model 2: model 1 with smoking status, drinking status, BMI, basic ADL, and instrumental ADL.
^c^ Model 3: model 2 with dyslipidemia, diabetes/high blood sugar, heart problems, cancer, chronic lung disease, memory-related disease, kidney disease, liver disease, arthritis, digestive disease, asthma, psychiatric problems.

Table S9. NRI and IDI statistics for new-onset stroke by elevated-CRP levels and hypertension in CAHRLS (2011-2015)

| Model | AUC |  | | NRI | | |  | | IDI | | |  |
| --- | --- | --- | --- | --- | --- | --- | --- | --- | --- | --- | --- | --- |
|  | Estimate | |  | | Estimate (95%CI) | *P* value | |  | | Estimate (95%CI) | *P* value | |
| All |  | |  | |  |  | |  | |  |  | |
| Conventional model^a^ | 0.7388 | |  | | Reference | _ | |  | | Reference | _ | |
| Conventional model +elevated-CRP levels | 0.7407 | |  | | -0.0038(-0.0961-0.0885) | 0.9642 | |  | | 0.0000(-0.0005-0.0006) | 0.9712 | |
| Conventional model +hypertension | 0.7393 | |  | | 0.1512(-0.0143-0.3166) | 0.0744 | |  | | 0.0000(-0.0004-0.0006) | 0.7657 | |
| Conventional model +elevated-CRP levels+ hypertension | 0.7636 | |  | | 0.3499(0.1980-0.5017) | <0.0001 | |  | | 0.0052(0.0018-0.0086) | 0.0026 | |

^a^: adjusted for age, sex, annual per-capita income, living place, education level, smoking status, drinking status, BMI, basic ADL, and instrumental ADL, dyslipidemia, diabetes/high blood sugar, heart problems, cancer, chronic lung disease, memory-related disease, kidney disease, liver disease, arthritis, digestive disease, asthma, psychiatric problems.
